# Bulk segregant analysis-sequencing and RNA-Seq analyses reveal candidate genes associated with albino phenotype in *Brassica napus*

**DOI:** 10.3389/fpls.2022.994616

**Published:** 2022-09-02

**Authors:** Shenhua Ye, Jie Yang, Yingying Huang, Jie Liu, Xiaowei Ma, Lun Zhao, Chaozhi Ma, Jinxing Tu, Jinxiong Shen, Tingdong Fu, Jing Wen

**Affiliations:** National Key Laboratory of Crop Genetic Improvement, College of Plant Science and Technology, National Center of Rapeseed Improvement in Wuhan, Huazhong Agricultural University, Wuhan, China

**Keywords:** albino, *Brassica napus*, BSA-Seq, chloroplast development, RNA-Seq

## Abstract

Inheritable albino mutants are excellent models for exploring the mechanism of chloroplast biogenesis and development. However, only a few non-lethal albino mutations have been reported to date in *Brassica* species. Here, we describe a resynthesized *Brassica napus* mutant, whose leaf, stem, and silique tissues showed an inheritable albino phenotype under field conditions after the bud stage but green phenotype in the greenhouse during the whole growing season, indicating that the albino phenotype depends on environmental conditions. Compared with the green leaves of the field-grown wild-type (GL) and greenhouse-grown mutant (WGL) plants, white leaves of the field-grown mutant (WL) showed significantly lower chlorophyll contents and structural defects in chloroplasts. Genetic analysis revealed that the albino phenotype of WL is recessive and is controlled by multiple genes. Bulk segregant analysis-sequencing (BSA-Seq) indicated that the candidate regions responsible for the albino phenotype spanned a total physical distance of approximately 49.68 Mb on chromosomes A03, A07, A08, C03, C04, C06, and C07. To gain insights into the molecular mechanisms that control chloroplast development in *B. napus*, we performed transcriptome (RNA-Seq) analysis of GL, WGL, and WL samples. GO and KEGG enrichment analyses suggested that differentially expressed genes (DEGs) associated with leaf color were significantly enriched in photosynthesis, ribosome biogenesis and chlorophyll metabolism. Further analysis indicated that DEGs involved in chloroplast development and chlorophyll metabolism were likely the main factors responsible for the albino phenotype in *B. napus*. A total of 59 DEGs were screened in the candidate regions, and four DEGs (*BnaC03G0522600NO*, *BnaC07G0481600NO*, *BnaC07G0497800NO*, and *BnaA08G0016300NO*) were identified as the most likely candidates responsible for the albino phenotype. Altogether, this study provides clues for elucidating the molecular mechanisms underlying chloroplast development in *B. napus*.

## Introduction

Chloroplast-defective mutants usually exhibit albino, variegated, striped, etiolated, pale-green, and zebra leaves (Jung et al., 2003; Qiao et al., 2011, 2013; Kato et al., 2012; Hayashi-Tsugane et al., 2014; Yang et al., 2015; Ye et al., 2016; Chen et al., 2019; Feng et al., 2019; Zhao et al., 2020b). Albino mutants with defective chloroplast development are valuable resources for understanding chloroplast biogenesis and development, but they are usually seedling lethal under natural growth conditions. Over the last two decades, considerable progress has been made in deciphering the molecular mechanisms underlying the white/albino leaf phenotype, and a large number of genes associated with chloroplast development have been isolated (Aluru et al., 2006; Yu et al., 2007; Yoo et al., 2009; Gong et al., 2013; Song et al., 2014; Pogson et al., 2015; Tang et al., 2017; Wang et al., 2017; Zhang et al., 2018, 2020; Cui et al., 2019). Chloroplast biogenesis and development are sensitive to environment, and plants can regulate chloroplast translation and protect chloroplasts from injury under low temperature stress. Several genes related to the temperature-sensitive albino phenotype of plants have been identified to date, such as *V3*, *St1* (Yoo et al., 2009), *OsV4* (Gong et al., 2014), *WLP1* (Song et al., 2014), *TCD11* (Wang et al., 2017), and *DUA1* (Cui et al., 2019). These genes perform different functions in chloroplast development. *V3*, and *St1* encode the subunits of ribonucleotide reductase, which can affect the expression of plastid genes encoding the transcription/translation apparatus. *OsV4* and *DUA1* encode chloroplast-targeted pentatricopeptide repeat (PPR) protein, which can bind to the plastid-encoded RNA polymerase (PEP) transcript and reduce the transcript levels of PEP genes. *WLP1* and *TCD11* encode the 50S ribosome L13 protein and the ribosomal small subunit protein S6, which are important components for chloroplast biogenesis. However, the molecular mechanisms that environment affects chloroplast development remain poorly elucidated.

Chloroplasts are important organelles that perform photosynthesis and serve as the production and storage sites of many plant hormones and metabolites (Pogson and Albrecht, 2011). Numerous studies have shown that the biogenesis and development of chloroplasts require cooperation between the nuclear-encoded RNA polymerase (NEP) and PEP, and are regulated by developmental and environmental signals (Jarvis and López-Juez, 2013). The transcription of PEP subunits (rpoA, rpoB, rpoC1, and rpoC2) is regulated by NEP (Liere et al., 2011). Most of the NEP is imported into the chloroplasts by translocons at the outer/inner envelope membranes of chloroplasts (TOC/TIC; Nakai, 2015). Chloroplast RNAs must be processed by a variety of NEP, especially PPR proteins. PPR proteins participate in posttranscriptional modifications in plants, such as RNA editing, splicing, stability, processing, translation, and maturation (Wang et al., 2021). Functionally defective PPR proteins usually result in abnormal plant development (Saha et al., 2007). GUN1, a PPR protein, impacts chloroplast development by regulating the tetrapyrrole biosynthesis and retrograde signaling pathways (Shimizu et al., 2019; Loudya et al., 2020). In rice, amino acid substitution in the PPR protein DUA1 led to the production of structurally abnormal chloroplasts and albino leaves (Cui et al., 2019). Moreover, the expression levels of some plastid-encoded ribosomal proteins that are essential for chloroplast development are also affected by PPR proteins, such as PPR2 (Williams and Barkan, 2003), PPR4 (Schmitz-Linneweber et al., 2006) and PPR5 (Beick et al., 2008).

Chlorophyll is the primary photosynthetic pigment, and its abundance in chloroplasts directly affects leaf color and photosynthetic efficiency. Most of the enzymes involved in biosynthesis of chlorophyll from glutamyl-tRNA have been well characterized (Jung et al., 2003; Tanaka and Tanaka, 2007; Masuda and Fujita, 2008; Zhao et al., 2020b). Additionally, phenotypes related to mutations in chlorophyll biosynthetic genes have also been identified in plants. For example, premature translational termination of the *OsPORB* transcript, which encodes a protochlorophyllide oxidoreductase, decreased the chlorophyll content of rice leaves below a certain threshold, resulting in severe degreening in leaves (Sakuraba et al., 2013b). Inactivation of three inactive Mg-chelatase subunits, CHLD, CHLI, and CHLH, decreased the chlorophyll content of plants and resulted in underdeveloped chloroplasts (Jung et al., 2003; Luo et al., 2013). Generally, the photosynthetic capacity of defective chloroplasts is lower than that of normal chloroplasts. The efficiency of photosynthetic reactions and energy metabolism in chloroplasts is determined by photosystem I (PSI), PSII, and photosynthetic electron transport (PET) chain (Foyer et al., 2012; Nelson and Junge, 2015). Psa and Psb are the core subunits of PSI and PSII, respectively. In addition, the light-harvesting chlorophyll protein complex (LHC) plays a crucial role in regulating the functions of the photosynthetic antenna system, and they can regulate the absorption of light energy and protect chloroplasts from light-induced damage (Johnson et al., 2011).

*B. napus* is the important oilseed crop in the world. Leaves of different colors exhibit different photosynthetic efficiencies, which directly affect the growth of *B. napus* plants, subsequently altering yield. Apart from their utility for studying chloroplast development and photosynthetic mechanism, the leaf color mutants of *B. napus* are also used as markers in breeding (Zhao et al., 2020a). In most studies conducted to date, mutants with abnormal chloroplasts generally exhibited yellow or variegated leaves (Wang et al., 2016a; Zhu et al., 2017; Zhao et al., 2020a,c; Zhang et al., 2021). Moreover, an albino mutant has not yet been reported in *B. napus*. Despite the focus on leaf color, the molecular mechanisms responsible for leaf color development in *B. napus* remain unknown. In this study, we characterized a resynthesized *B. napus* mutant line, which exhibits albino phenotype in the field and normal (green) phenotype in the greenhouse. BSA-Seq and RNA-Seq analyses of green and albino plants revealed a large number of genes associated with chloroplast development in *B. napus*. These results provide a foundation for understanding the molecular mechanisms of chloroplast development in *B. napus*.

## Materials and methods

### Plant materials and growth conditions

**Figure 1:**
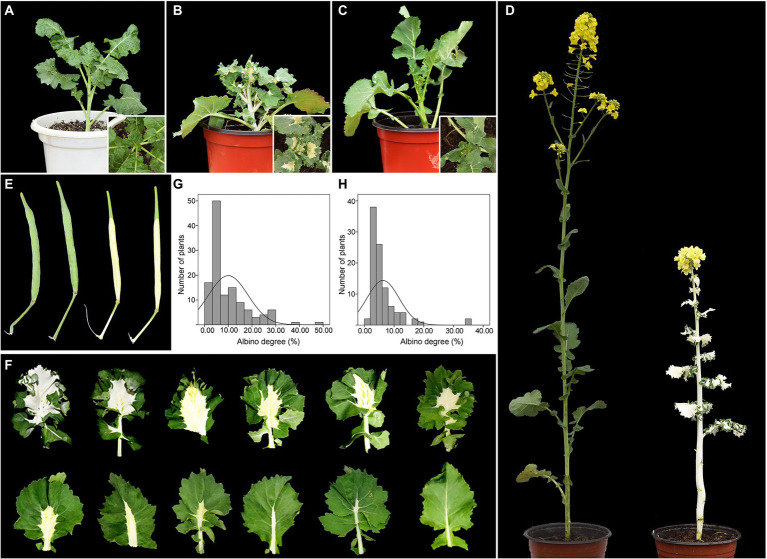
Phenotypic characterization of *B. napus* lines G7097 and W7105. **(A)** Field-grown G7097 (GL). **(B)** Field-grown W7105 (WL). **(C)** Greenhouse-grown W7105 (WGL). **(D)** Field-grown GL (left) and WL (right) at the flowering stage. **(E)** Siliques of GL (left) and WL (right). **(F)** Phenotype of the leaves of BC_1_F_2_ plants. **(G)** Frequency distribution of albino-part/leaf area ratio in the BC_1_ population (*n* = 124). **(H)** Frequency distribution of albino-part/leaf area ratio in the BC_1_F_2_ population (*n* = 97).

One white-leaf (W7105) and two green-leaf (G7097 and 2,127) *B. napus* lines were used in this study. G7097 is a resynthesized *B. napus* line that was developed by interspecific hybridization between *Brassica oleracea* and *Brassica rapa* (Wen et al., 2008), followed by self-pollination over six flowering seasons. W7105 is a spontaneous mutant isolated from G7097. 2,127 is a green-leaf resynthesized *B. napus* line originated from *B. alboglabra* and *B. rapa* (Chen et al., 1988). Reciprocal crosses were performed between W7105 and 2,127. The resulting F_1_ progeny was then back-crossed with W7105 to produce the BC_1_ segregating population. Green-leaf plants in the BC_1_ segregating population were self-pollinated to produce the BC_1_F_2_ segregating population. G7097 (hereafter designated as GL; Figure 1A), W7105 (hereafter field-grown W7105 was designated as WL; Figure 1B), 2,127, and their progenies were grown in an open field in Huazhong Agricultural University.

The ambient temperature during growing season is summarized in Supplementary Table S1. To study whether the albino phenotype of field-grown W7105 plants was induced by different environment, 10 seedlings of W7105 (hereafter greenhouse-grown W7105 was designated as WGL; Figure 1C) grown in the field for 30 days were transferred into a greenhouse and grown at 25°C under 16 h light/8 h dark photoperiod at all development stages. Phenotypes of greenhouse-grown plants were then compared with those of their field-grown counterparts.

### Trait evaluation

At the early flowering stage, the average albino degree of the newest three leaves was investigated in BC_1_ and BC_1_F_2_ individuals which were derived from the crosses between W7105 and 2,127. Albino degree was defined as the ratio of albino area to the whole leaf area. Albino area and leaf area were obtained using the methods described by Perez et al. (2000). To understand the impact of albino phenotype on plant reproduction, five representative plants of WL and GL lines were used for trait evaluation. As previously described (Ding et al., 2012; Li et al., 2020), eight yield-related traits were evaluated, including plant height, main inflorescence length, number of primary branches, silique number per plant, silique length, seed number per silique, thousand-seed weight and seed yield per plant. The data were analyzed *via* Microsoft Excel using two tailed Student’s *t*-test analysis.

### Measurement of chlorophyll and carotenoid contents

At the bud stage, the chlorophyll and carotenoid contents of newly emerged leaves of GL, WL, and WGL lines were determined, according to the method described by Zhu et al. (2017). A 0.2 g fresh leaf sample of each line was immersed in 10 ml of 80% acetone for 48 h at 4°C in the dark, following which plant debris was removed by centrifugation at 5,000 × *g* for 10 min. The supernatant was analyzed by spectrophotometric scanning at 665, 649, and 470 nm, with 80% acetone as the control. The concentrations of chlorophyll a (Chl a), chlorophyll b (Chl b), and carotenoids (Car) were calculated according to the following equations (Arnon, 1949):


$$\mathrm{Chlorophyll}\;a:\mathrm{Chl}\;a=12.21A_{663-}2.81A_{646}.$$

$$\mathrm{Chlorophyll}\;b:\mathrm{Chl}\;b=20.13A_{646-}5.03A_{663}.$$

$$\mathrm{Carotenoid}:\mathrm{Car}=\left(1000A_{470-}3.27\mathrm{Chl}\;a-10\mathrm{4Chl}\;b\right)/229.$$

Three biological replicates were performed for each line, with each replicate containing three technical repeats.

### Transmission electron microscopy assay

Young leaves were cut into 1 mm × 1 mm sections, and fixed in 2.5% glutaraldehyde in 0.2 M phosphate-buffered saline (PBS, pH 7.2) under vacuum. After overnight incubation at 4°C, the samples were rinsed three times with 0.2 M PBS for 10 min each time. Then, the samples were fixed in 1% osmium tetroxide in 0.1 M PBS for approximately 2 h, and then washed with 0.2 M PBS as described above. After washing, the samples were dehydrated with a graded acetone series (30, 50, 70, 80, 90, and 100%; 30 min at each concentration), and embedded in Polybed 812 (Epon) resin. The embedded specimens were sliced using ultramicrotome Leica UC6, and sections were stained with 2% (*w*/*v*) uranyl acetate and 2.6% (*w*/*v*) lead citrate for 10 and 5 min, respectively. Finally, the dried ultrathin sections were observed and photographed using a 100-kV H-7650 Transmission Electron Microscope (Hitachi, Tokyo, Japan), equipped with a digital camera.

### Bulk segregant analysis-sequencing

To perform BSA-Seq, leaf samples at the bud stage were collected from green-leaf and white-leaf individuals in the BC_1_F_2_ generation, and genomic DNA was extracted using the Hi-DNA Secure Plant Kit (Tiangen, Beijing, China). The quality of DNA was assessed using NanoDrop 2000 spectrophotometer (Thermo Scientific, MA, United States). The DNA samples of 30 individuals of each phenotype were mixed in equal quantities to obtain a green pool and a white pool. 2,127 and WL were used as parental pools. Four DNA libraries were constructed, and sequenced on the Illumina HiSeq X Ten platform (Illumina, CA, United States). Raw data were filtered using the fastp software to remove low-quality reads and adapter sequences (Chen et al., 2018). HISAT2 was used to map the reads to the reference genome of *B. napus* 2,127 (Kim et al., 2019).^1^ Single nucleotide polymorphisms (SNPs) and insertion/deletion mutations (InDels) were conducted using the Genome Analysis Toolkit (GATK) software (McKenna et al., 2010). Sliding window analysis was used to calculate the average distribution of SNPs across the 19 chromosomes of *B. napus*, with 1 Mb window size and 100 kb increment. The value of Δ(SNP index) was calculated as the difference in SNP indices between the two pools, as described previously (Guo et al., 2020). Candidate regions were selected based on 95% confidence intervals (Takagi et al., 2013).

### RNA extraction and cDNA library construction

Total RNA was extracted using the TRIzol kit (Invitrogen, CA, United States) from newly emerged leaves harvested from GL, WGL, and WL lines at the bud stage, and purified using mRNA purification kit (Promega, WI, United States), according to the manufacturer’s instructions. With three biological replicates per sample, nine cDNA libraries were constructed using the Illumina TruSeq RNA Sample Preparation Kit (Illumina, CA, United States), according to the manufacturer’s instructions. The concentration and quality of each cDNA library were assessed using Agilent 2,100 Bioanaylzer (Agilent Technologies, CA, United States) and ABI Step One Plus Real-Time PCR System (Applied Biosystems, CA, United States), respectively. Then, the libraries were sequenced on the Illumina HiSeq X Ten platform (Illumina, CA, United States).

### RNA-Seq data analysis

Raw RNA-Seq data were processed to remove low-quality reads and reads containing adapter sequences and high content of unknown bases (Ns). The resultant clean reads of each sample were mapped to the reference genome sequence of *B. napus* 2,127 by HISAT2 (Kim et al., 2019).^2^ Only uniquely mapped reads were considered for gene expression analysis. Differential gene expression and transcript abundance (expressed as Fragments Per Kilobase per Million mapped reads (FPKM) values) were calculated using the RESM program (Li and Dewey, 2011). Genes with FPKM < 1 in all samples were excluded from subsequent analysis. Differentially expressed genes (DEGs) were identified using DESeq2 based on two criteria: false discovery rate (FDR) < 0.01 and |log_2_fold change (FC)| > 1 (Love et al., 2014).

### Functional annotation of DEGs

Gene Ontology (GO) and Kyoto Encyclopedia of Genes and Genomes (KEGG) functional annotations for DEGs were retrieved using blast2go^3^ and blastx/blastp searches against the GO database^4^ and KEGG database,^5^ respectively. GO terms with *p* values ≤ 0.0001 and KEGG pathways with *Q*-values ≤ 0.05 were considered to be significantly significant.

### Quantitative real-time PCR validation

Total RNA extracted for transcriptome sequencing was used for conducting qRT-PCR. Eight genes relevant to chlorophyll synthesis and chloroplast development were selected to validate RNA sequencing data by qRT-PCR. The primer pairs were designed using Primer 5.0 (Thermo Fisher, MA, United States). Details of primer pairs are presented in supplementary Table S2. qRT-PCR was performed on an ABI StepOne^™^ Real-time PCR System (Applied Biosystems, CA, United States). All qRT-PCR experiments included three technical replicates and three biological replicates. The *B. napus actin7* gene was used as an internal control.

## Results

### Characterization and genetic analysis of the albino mutant

Under field conditions, the GL line G7097 produced green leaves throughout the growth period, whereas field-grown W7105 (WL) showed leaf color variation at the bud stage (Figures 1A,B). During the period from the early October to late January, when daily mean temperatures gradually dropped below 10°C for more than 2 months (Supplementary Table S1), leaves of WL seedlings keep green until the seven-leaf or eight-leaf stage stages. Then the newly emerged WL leaves showed albino phenotype from the eight or nine-leaf stage onward, and leaves that were already fully expanded maintained their green color. Interestingly, all the white leaves of WL seedlings emerged under field conditions had green leaf margins, and the albino area extended from the midrib to the peripheries (Figure 1B). Additionally, WL also exhibited white stems and white siliques (not including the silique beak; Figures 1D,E). By contrast, each leaf of greenhouse-grown W7105 (WGL), the counterpart of WL, remained green at a temperature of approximately 25°C in the greenhouse (Figure 1C). The results indicated that the albino phenotype of W7105 is depend on different environment. A period of low temperature plausibly changed the phenotype from normal to albino in WL. To study the impact of albinism on plant development, eight agronomic traits were investigated for WL and GL lines (Supplementary Table S3). The results showed that GL had significantly higher plant height and main inflorescence length in compared to WL. Importantly, several yield-related traits of GL, including silique number per plant, silique length, seed number per silique was about 1.53–2.61-fold higher than those of WL. The average seed yield per plant of WL was only 2.3 g, representing only 16.3% of GL, indicating white leaves, stems, and siliques of *B. napus* inevitably resulted in reduced photosynthetic capacity and plant yield.

To further determine the inheritance pattern of the albino phenotype, we generated F_1_, BC_1_, and BC_1_F_2_ populations using W7105 and green-leaf 2,127 as parents. All F_1_ plants exhibited green leaves, stems, and siliques. In the BC_1_ and BC_1_F_2_ population, the leaves of plants exhibited varying degrees of albino phenotype (Figure 1F). The frequency of albino degree followed a skewed distribution pattern (Figures 1G,H), indicating that the albino phenotype of W7105 is a recessive trait and is controlled by multiple genes.

### Pigment contents of green and white leaves

**Figure 2:**
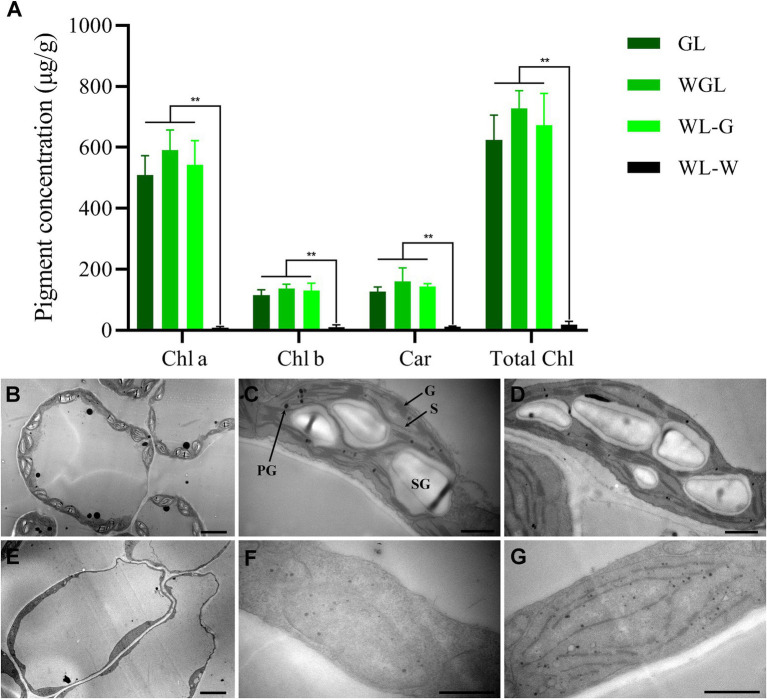
Pigment contents and transmission electron micrographs of the young leaves of GL, WGL, and WL plants. **(A)** Pigment contents of young leaves at the bud stage. Chl a, chlorophyll a; Chl b, chlorophyll b; Car, carotenoid; Total Chl, total chlorophyll. Error bars represent the standard deviation (SD) of three biological replicates. Asterisks indicate statistically significant differences (^**^p < 0.01; Student’s *t*-test). **(B)** Chloroplasts in WGL mesophyll cells. Scale bar = 5 μm. **(C,D)** Ultrastructure of chloroplasts in GL leaf **(C)** and the green sectors of WL leaf **(D)**. Scale bars = 1 μm. **(E)** Chloroplasts in mesophyll cells of WL albino leaf sectors. Scale bar = 5 μm. **(F,G)** Ultrastructure of chloroplasts in albino sectors of WL. Scale bar = 1 μm. PG, plastoglobule; G, grana thylakoid; S, stroma thylakoid; SG, starch granule.

To determine whether the albino phenotype of WL was caused by a defect in pigment accumulation, the chlorophyll and carotenoid contents of GL, WL, and WGL leaves were measured (Figure 2A). In young leaves collected from WL plants, pigment contents of the white main part (WL-W) and green leaf margin (WL-G) were investigated separately. As expected, the levels of chlorophyll a, chlorophyll b, and carotenoids were higher in the green leaves of GL and WGL plants and in WL-G. The total chlorophyll contents of GL, WGL, and WL-G were 624.32, 728.26, and 671.93 μg/g, respectively. The carotenoid contents of GL, WGL, and WL-G were 126.40, 160.15, and 143.9 μg/g, respectively. No significant differences were detected in chlorophyll a, chlorophyll b, total chlorophyll, and carotenoid contents among the green tissues of GL, WGL, and WL-G. However, nearly no chlorophyll and carotenoid accumulations were detected in WL-W. These results suggested that the albino phenotype of WL is caused by a dramatic reduction in chlorophyll content.

### Ultrastructural analysis of leaf chloroplasts

To examine whether the lack of chlorophyll in the albino part of WL leaves was accompanied by ultrastructural changes in chloroplasts, we analyzed the electron micrographs of ultrathin sections of leaves (Figures 2B–G). In the green leaves of GL and WGL, chloroplasts are crescent-shaped with numerous well-developed grana thylakoids and unstacked stroma thylakoids, and shuttle-shaped starch granules were frequently observed in these chloroplasts. Consistent with the results of chlorophyll contents, the green sectors of WL leaves showed normal chloroplasts, similar to those observed in GL and WGL leaves. However, the albino part of WL leaves showed linear-shaped chloroplasts with no starch granules. In most cases, the chloroplasts were completely devoid of internal membrane structures, including stromal thylakoids and stacked grana thylakoids. Nonetheless, some chloroplasts contained internal membrane systems, albeit poorly developed. In these chloroplasts, the grana could not be stacked normally, and grana and stroma thylakoids were linear. Additionally, compared with green leaves, plastoglobules (PGs) were more abundant in the chloroplasts of WL white leaves. PGs often accumulate in plastid loss-of-function mutants, particular those with defects in thylakoid formation (Wijk and Kessler, 2017). These results collectively indicated that the lack of chlorophyll in the albino part of WL leaves was accompanied by defects in chloroplast development, and harsh environment compromised chloroplast development in WL leaves.

### BSA-Seq analysis

**Figure 3:**
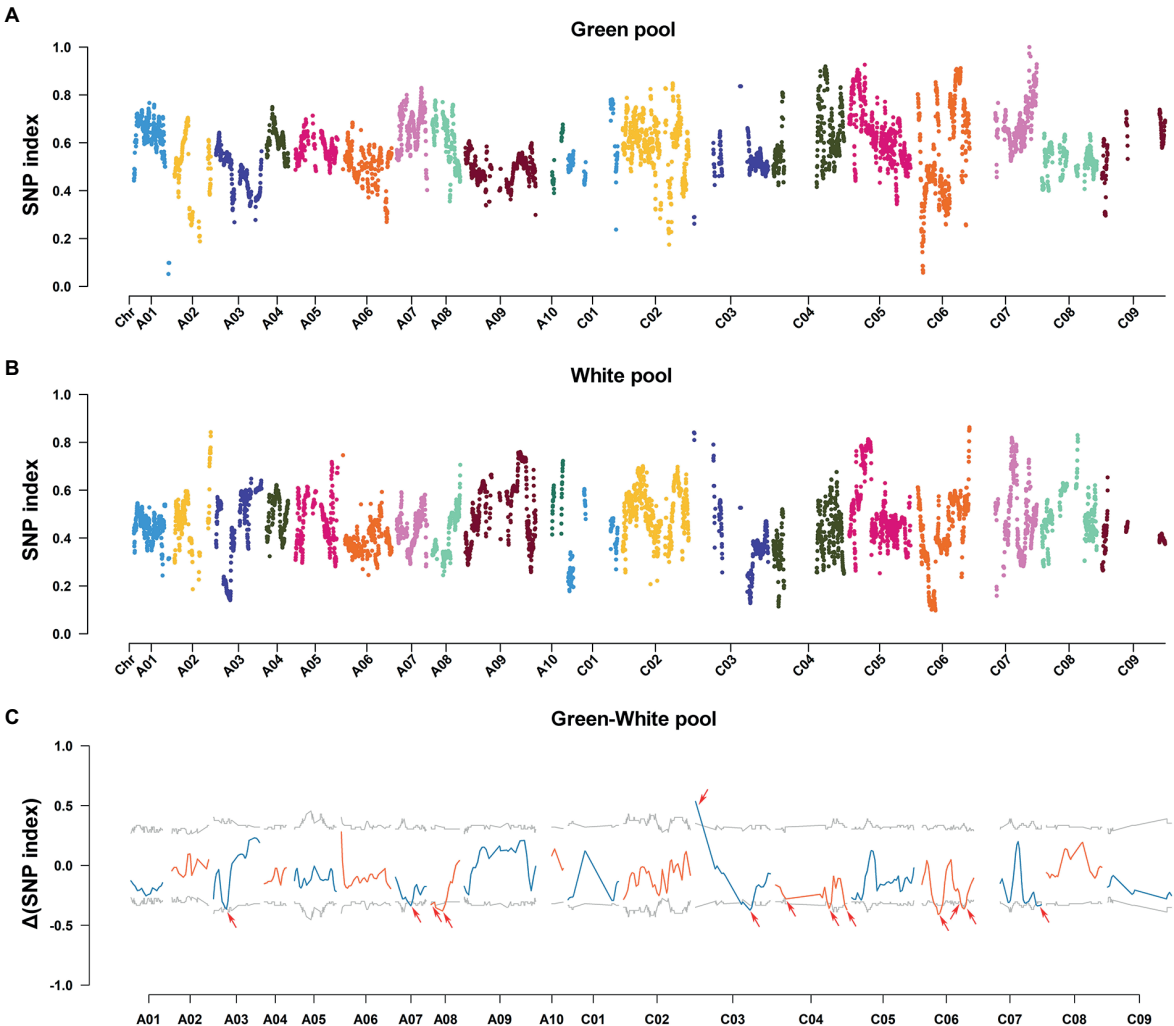
Single nucleotide polymorphism-index and Δ(SNP-index) in BSA-seq analysis. **(A,B)** SNP index distribution of the green pool **(A)** and white pool **(B)** in the BC_1_F_2_ population. **(C)** Δ(SNP-index) plot of the green and white pools. Red arrows indicate candidate regions.

To identify the candidate genes associated with albino phenotype of WL, BSA-Seq analysis was performed using BC_1_F_2_ plants exhibiting extreme leaf color phenotype as materials. After filtration, we obtained a total of 114.60 Gb clean data, which included 37.04, 39.21, 17.29, and 21.06 Gb corresponding to the green pool, white pool, 2,127 parental pool, and WL parental pool (Supplementary Table S4). The Q30 ratio and GC content of each pool was greater than 94.90% and greater than 37.37%, respectively. A total of 375,283 SNPs were identified in the green pool and white pool. Based on Δ(SNP-index), a total of 13 candidate regions were identified on chromosomes A03, A07, A08, C03, C04, C06, and C07 (Figure 3; Supplementary Table S5). Together, these candidate regions spanned a physical distance of approximately 49.68 Mb and contained 4,390 annotated genes.

### RNA-Seq assembly, unigene annotation, and DEG analysis

To gain insights into the genetic and regulatory mechanisms underlying the temperature-sensitive albino phenotype, the newly emerged leaves of GL, WL, and WGL lines were harvested in three independent biological replicates at the bud stage and subjected to transcriptome sequencing. After filtering the raw data, a total of 18.59, 18.77, and 18.70 Gb clean data were obtained for GL, WL and WGL, respectively (Supplementary Table S6). More than 70% of these clean reads were mapped to unique genomic locations, and the uniquely mapped reads were used for further analysis. Pearson correlation coefficient analysis of three replicates exhibited consistency, indicating that the RNA-Seq results were highly reliable (Supplementary Figure S1).

**Figure 4:**
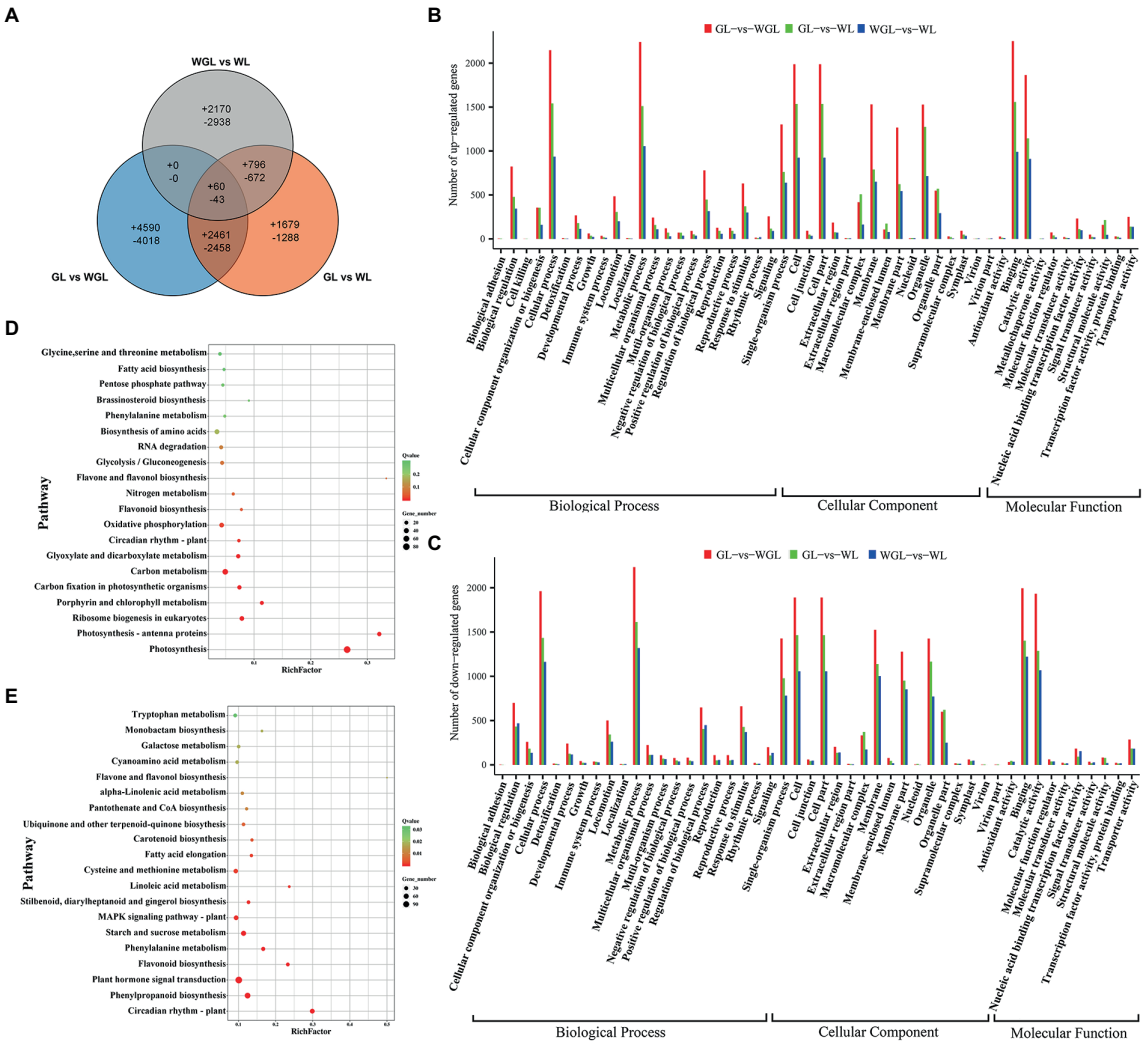
Analysis of DEGs identified among GL, WL, and WGL leaves. **(A)** Venn diagram showing the DEGs unique and common to the three comparisons (GL *vs*. WL, GL *vs*. WGL, and WGL *vs*. WL). **(B,C)** GO classifications of up-regulated **(B)** and down-regulated **(C)** genes. **(D)** KEGG pathway enrichment analysis of DEGs common to GL *vs*. WL and WGL *vs*. WL comparisons. **(E)** KEGG pathway enrichment of DEGs unique to WGL *vs*. WL comparison.

A total of 58,531, 61,087, and 61,767 expressed genes were identified in the leaves of GL, WL, and WGL, respectively. Then, three pairwise comparisons of gene transcript levels were performed: GL (G7097) *vs*. WL (W7105), to compare near isogenic lines (NILs) that differed in leaf color in the field; WGL *vs*. WL, to compare W7105 grown in the greenhouse (green leaves) with W7105 grown in the field (white leaves); GL (G7097) *vs*. WGL (W7105), to compare NILs that exhibited the same green leaf color in different growing environments. The distribution of DEGs uniquely expressed in each comparison and that of DEGs expressed in two or more comparisons are shown in Figure 4A. In comparison with GL, we identified 9,457 DEGs (4,996 up-regulated and 4,461 down-regulated) in WL, and 13,630 DEGs (7,111 up-regulated and 6,519 down-regulated) in WGL. In comparison with WGL, we found 3,026 up-regulated and 3,653 down-regulated genes in WL.

### Gene ontology and KEGG enrichment analyses of DEGs

To clarify the functional significance of DEGs, further GO and KEGG enrichment analysis were performed. All the above-mentioned DEGs were successfully assigned to three main GO categories (Figures 4B,C). Metabolic process, cell, cell part, cellular process, and binding were the five most common GO terms in all three comparisons mentioned above. In the GL *vs*. WGL comparison, GO terms related to plant response and chloroplast structure, such as response to abiotic stimulus, response to oxygen-containing compound, and thylakoid part, were the most highly represented. GO terms related to chloroplast structure and photosystem, such as thylakoid, thylakoid membrane, photosynthetic membrane, photosystem, showed the highest enrichment ratios in comparisons between green leaves and white leaves (GL *vs*. WL and WGL *vs*. WL; Supplementary Figure S2).

By and large, the KEGG pathway enrichment analysis supported the GO classification. The genes down-regulated in WGL compared with GL were mostly enriched in pathways for circadian rhythm, photosynthesis-antenna proteins, and flavonoid biosynthesis, while the up-regulated genes were significantly enriched in cysteine and methionine metabolism, MAPK signaling pathway, and ribosome (Supplementary Figures S3A,B). Compared with GL, the KEGG pathways of photosynthesis, photosynthesis-antenna protein, and carbon fixation in photosynthetic organisms were significantly enriched among the genes down-regulated in WL, while the pathways of ribosome, ribosome biogenesis in eukaryotes, and RNA degradation were significantly enriched among genes up-regulated in WL (Supplementary Figures S3C,D). In WGL *vs*. WL, the most highly enriched pathways involving down-regulated genes in WL were photosynthesis, photosynthesis-antenna protein, and glyoxylate and dicarboxylate metabolism, and those involving up-regulated genes were circadian rhythm, flavonoid biosynthesis, and phenylpropanoid biosynthesis (Supplementary Figures S3E,F). KEGG enrichment analysis of DEGs identified in GL *vs*. WL and WGL *vs*. WL comparisons revealed that the pathways of ribosome biogenesis in eukaryotes, porphyrin and chlorophyll metabolism, and carbon fixation in photosynthetic organisms were vital and common pathways that determined or were affected by defects in chloroplast development (Figure 4D). In addition, pathways associated with photosynthesis, such as photosynthesis, photosynthesis-antenna proteins, oxygenic photosynthesis, Calvin cycle, and photorespiration, and those associated with other metabolic processes that take place in chloroplasts, such as amino acid biosynthesis and starch and sucrose metabolism, were also significantly changed. DEGs uniquely identified in the WGL *vs*. WL comparison were mostly enriched in circadian rhythm, phenylpropanoid biosynthesis, and plant hormone signal transduction (Figure 4E), indicating the important roles of these pathways in environment-induced leaf color transition.

### Putative DEGs related to chloroplast development, chlorophyll metabolism and photosynthesis

Chloroplast development is regulated by the coordinated expression of nuclear-encoded and plastid-encoded genes, especially ribosomal proteins and PPR proteins. As expected, we identified 30 DEGs related to ribosome (ko03010), which were common between GL *vs*. WL and WGL *vs*. WL comparisons (Supplementary Table S7). Further investigation revealed that the expression levels of 86.67% of these DEGs were significantly higher in WL than in GL and WGL. Among the 27 DEGs encoding chloroplast PPR proteins (Supplementary Table S7), only four genes (*BnaC05G0527400NO*, *BnaC07G0208600NO*, *BnaC08G0027900NO*, and *BnaA08G0016300NO*) were down-regulated. The highly expressed PPR gene, *BnaC03G0102300NO (GUN1)*, which is responsible for retrograde signaling during chloroplast development, was up-regulated by 2.29- to 3.42-fold in WL. Moreover, the expression levels of *BnaC04G0338800NO* and *BnaC02G0358400NO (ropA)*, which are required for initiating chloroplast biogenesis, were up-regulated by 4.54- to 9995.98-fold in WL (Supplementary Table S7). These results indicated that the abovementioned DEGs play critical roles in chloroplast development in *B. napus*.

**Figure 5:**
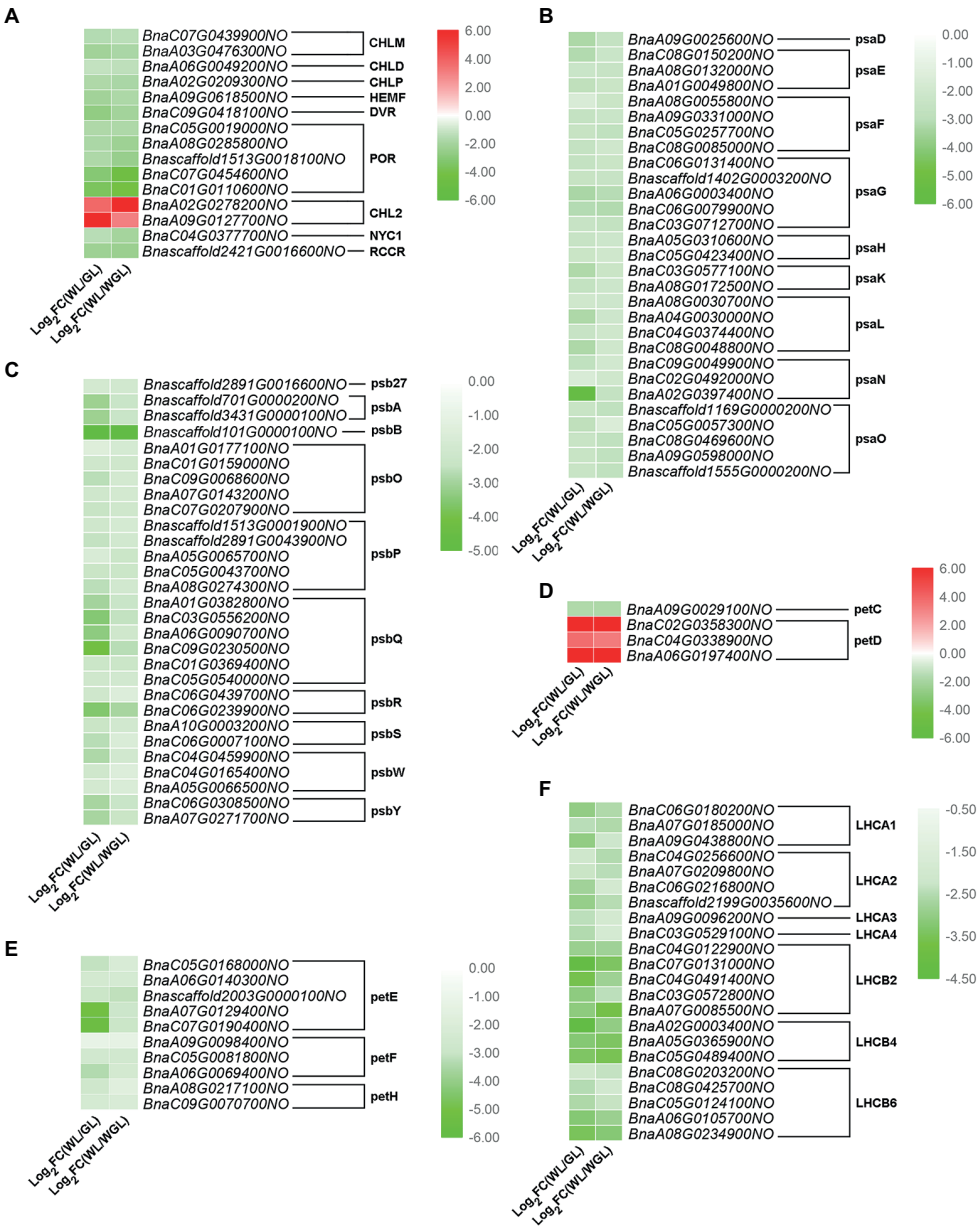
Expression differences of DEGs related to chlorophyll metabolism and photosynthesis in the comparison of GL *vs*. WL and WGL *vs*. WL. **(A–F)** Expression differences of DEGs related to porphyrin and chlorophyll metabolism **(A)**, photosystem I **(B)**, photosystem II **(C)**, cytochrome b6/f complex **(D)**, photosynthetic electron transport **(E)**, and light-harvesting chlorophyll protein complex **(F)** in the comparison of GL *vs*. WL and WGL *vs*. WL. The red to green gradient in the heatmaps indicates log_2_ (fold change) values from high to low, respectively.

Chloroplast development is closely related to chlorophyll metabolism and photosynthesis. Therefore, DEGs related to porphyrin and chlorophyll metabolism were analyzed in detail. Nearly all DEGs involved in porphyrin and chlorophyll metabolism were down-regulated in white leaves, except for two DEGs (*BnaA02G0278200NO* and *BnaA09G0127700NO*), both of which encode chlorophyllase-2 (CHL2; Figure 5A). In the photosynthesis pathway, the transcript levels of all DEGs encoding PSI and PSII core subunits (psaD, psaE, psaF, psaG, psaH, psaK, psaL, psaN, psaO, psb27, psbA, psbB, psbO, psbP, psbQ, psbR, psbS, psbW, and psbY) were significantly reduced (Figures 5B,C). Among the DEGs related to cytochrome b6/f complex and photosynthetic electron transport, only three DEGs (*BnaC02G0358300NO*, *BnaC04G0338900NO*, and *BnaA06G0197400NO*) encoding petD proteins were up-regulated, while those encoding other subunits (petC, petE, petF, and petH) were down-regulated (Figures 5D,E). In addition, all DEGs (*LHCA1*, *LHCA2*, *LHCA3*, *LHCA4*, *LHCB1*, *LHCB4*, and *LHCB6*) encoding light-harvesting chlorophyll protein complex were down-regulated by 3.54- to 21.16-fold in white leaves relative to green leaves (Figure 5F). These results indicated that the defects in chloroplast development and chlorophyll metabolism result in the down-regulation of a large number of photosynthesis-related DEGs in WL.

### Putative DEGs related to carotenoid biosynthesis

As a component of photosynthetic pigments, carotenoids play a crucial role in photoprotective functions (Tanaka et al., 2008). Since the carotenoid content in the white leaves was significantly lower than that in the green leaves, we then focused on the expressional changes of carotenoid synthesis genes. As expected, three DEGs (*BnaC02G0031000NO*, *BnaA06G0049500NO*, and *BnaC05G0058700NO*) related to carotenoid biosynthesis (ko00906) were identified (Supplementary Table S8), which were common between GL *vs*. WL and WGL *vs*. WL. Compared with green leaves, *BnaC02G0031000NO* encoding phytoene synthase (crtB), and *BnaA06G0049500NO* and *BnaC05G0058700NO* encoding violaxanthin de-epoxidase (VDE), were down-regulated by 2.46- to 32.56-fold in white leaves. These results indicated that the down-regulated *crtB* and *VDE* genes might lead to the reduced carotenoid content in WL.

### Association analysis between BSA-Seq and RNA-Seq data

To rapidly identify candidate genes associated with the albino phenotype of WL, association analysis was performed by combining the BSA-Seq and RNA-Seq results. Based on the transcriptomic data, a total of 59 DEGs were screened out in the candidate regions associated with the white-leaf phenotype (Supplementary Table S9). Among these, four DEGs including *BnaC03G0522600NO* (*TOC75-3*), *BnaC07G0481600NO* (*TIC62*), *BnaC07G0497800NO* (*arginine-tRNA synthetase* gene), and *BnaA08G0016300NO* (PPR protein gene, *DYW1*), which potentially participate in chloroplast development (Ling et al., 2009; Nakai, 2015; Li et al., 2021), were identified as the most likely candidates responsible for the albino phenotype of WL.

### qRT-PCR analysis

To validate the reliability of the RNA-Seq analysis and the expression profiles of the important homologs related to chlorophyll biosynthesis and photosynthesis, qRT-PCR analysis was conducted to analyze the expression of eight selected genes (*BnaC01G0110600NO*, *POR*; *BnaC06G0180200NO*, *LHCA1*; *Bnascaffold2199G0035600NO*, *LHCA2*; *BnaA05G0365900NO*, *LHCB4*; *BnaC05G0081800NO*, *petF*; *Bnascaffold2003G0000100NO*, *petE*; *BnaC09G0230500NO*, *psbQ*; *BnaC06G0079900NO*, *psaG*). The results showed that the express patterns of the genes determined by qRT-PCR were consistent with those identified by RNA-Seq (Supplementary Figure S4), suggesting the reliability of transcriptional expression results in this study.

## Discussion

In higher plants, chloroplast development has been a key focus of research. Limited information is available on chloroplast development and the underlying regulatory mechanisms, especially in *Brassica* species. In kale (*B. oleracea*), a temperature-sensitive chlorophyll mutant called “white dove” was identified, which exhibited an albino phenotype in the interior of the plant under low temperature conditions (Zhou et al., 2013). A recent study reported that the cool-temperature-induced albinism of ornamental kale was controlled by one semi-dominant gene, which was mapped to an approximately 60 kb interval on chromosome C03 (Yan et al., 2020). To the best of our knowledge, W7105 is the first albino mutant reported in *B. napus*. W7105 differs from seedling lethal mutants, as it can survive and produce progeny under field conditions. The albino phenotype of W7105 may be induced by a period of low temperature, and it did not revert back to green phenotype under field conditions after the bud stage when the temperature gradually increases. Therefore, W7105 is an excellent resource for understanding the molecular mechanisms of chloroplast development and for investigating how environment affects chloroplast development in *B. napus*.

### Putative genes for chloroplast development in *Brassica napus*

Bulk segregant analysis-sequencing (BSA-Seq) is a good approach to rapidly identify candidate regions and help accurately map the candidate genes in various plants. This approach has been successfully used to map the genes for various quantitative traits, such as fruit color (Oren et al., 2019), fruit texture (Wu et al., 2021) and grain length (Wang et al., 2022). In this study, a total of 13 candidate regions, associated with the temperature-sensitive albino phenotype of *B. napus*, were identified using BSA-Seq. Combined with RNA-Seq analysis, four genes encoding TOC75-3 (*BnaC03G0522600NO*), TIC62 (*BnaC07G0481600NO*), arginine-tRNA synthetase (*BnaC07G0497800NO*), and PPR protein DYW1 (*BnaA08G0016300NO*), were identified as the most likely candidate genes that potentially associated with chloroplast development. TOC75-3 and TIC62 are component of the TOC/TIC complex. Growing evidence indicate that the TOC/TIC complex participates in plastid biogenesis through directly regulating accompany proteome remodeling (Richardson and Schnell, 2020). Arginine-tRNA synthetase is one of the aminoacyl-tRNA synthetases and may affect chloroplast development and ribosomal biogenesis. Previous studies showed that the dysfunction of the Val-tRNA synthetase and the glyctl-tRNA synthetase gene, which play important roles in ribosomal biosynthesis, resulted in defective chloroplast development and albino leaves at seedling stage in rice (Wang et al., 2016b; Zheng et al., 2019). PPR protein DYW1 contains a conserved DYW domain and is necessary for editing of *ndhD-1* (Boussardon et al., 2014). However, the functions of the above candidate genes have not been validated in *B, napus*, and further experiments will help clarify the involvement of these candidate genes in chloroplast development.

### Abnormal chloroplast development led to *Brassica napus* albinism

Leaf color is directly determined by pigment accumulation in chloroplasts. Thus, abnormal chloroplast development or defective chlorophyll metabolism will lead to color change in leaves (Pogson et al., 2015). In the unique example of an albino mutant in *Brassica* crops, the protoplasts of albino kale tissues had abnormal chloroplasts, exhibiting defective grana thylakoids (Yan et al., 2020). In this study, the transmission electron microscope observation in albino leaves of WL showed that most of the chloroplasts were poorly developed and devoid of internal membrane structures (Figures 2B–G). Accordingly, a number of genes related to chloroplast development were significantly differentially expressed in WL when compared to GL and WGL, indicating defects in chloroplast development were responsible for the albino phenotype in *B. napus*. Plastid gene transcription requires strict coordination among PEP, NEP, and GUN1-mediated retrograde signaling (Tadini et al., 2020). In this study, *BnaC04.rpoA* (*BnaC04G0338800NO*), *BnaC02.rpoA* (*BnaC02G0358400NO*), and *BnaC03.GUN1* (*BnaC03G0102300NO*) were up-regulated in WL, which may directly affect plastid transcription, resulting in defective chloroplast development. PPR proteins play crucial functions in regulating organelle gene expression (Saha et al., 2007). For example, Arabidopsis DG1 and YS1 are both chloroplast-targeted PPR proteins, and YS1 was characterized by editing *rpoB* transcripts. The mutation of *DG1* and *YS1* genes in Arabidopsis led to an albino and a yellow seedling phenotype, respectively (Chi et al., 2008; Zhou et al., 2009). Similarly, essential ribosomal proteins edited by PPR proteins are required for chloroplast development. Any defects in these ribosomal proteins such as RPS17 (Schultes et al., 2000), ASL1 (Gong et al., 2013), ASL2 (Lin et al., 2015), RPL12 (Zhao et al., 2016), RPL13 (Song et al., 2014), and TCD11 (Wang et al., 2017) led to abnormal chloroplast development. Apart from *rpoA* and *GUN1*, we also found that a majority of genes encoding ribosomal and PPR proteins were up-regulated in WL (Supplementary Table S5). These genes may also play critical roles in chloroplast development in *B. napus*.

Chloroplast biogenesis and development were usually accompanied by chlorophyll accumulation (Pogson et al., 2015). Plants with defective chloroplasts usually exhibit a defect in chlorophyll accumulation. In support of this, our results showed that the levels of chlorophyll contents were significantly higher in the green leaves of GL and WGL plants when compared to WL (Figure 2A). In agreement with the chlorophyll results, the key genes involved in chlorophyll metabolism, including *magnesium protoporphyrin IX methyltransferase* (*CHLM*)*, CHLD*, *inactivation of the geranylgeranyl reductase* (*CHLP*), *oxygen-dependent coproporphyrinogen III oxidase* (*HEMF*), *8-vinyl-reductase* (*DVR*), *protochlorophyllide oxidoreductase* (*POR*), *non-yellow coloring 1* (*NYC1*), and *red chlorophyll catabolite reductase* (*RCCR*), and significantly down-regulated in WL (Figure 5A). Mg-chelatase consists of three subunits (CHLH, CHLI and CHLD) and participates in the insertion of magnesium into protoporphyrin IX in chlorophyll metabolism (Willows et al., 1996). A single amino acid change of CHLD altered chloroplast ultrastructure and chlorophyll accumulation, resulting in the yellow-green leaf in foxtail millet (Li et al., 2016). Previous studies suggested that *POR* is essential for chlorophyll synthesis, and when *PORB* and *PORC* mutated, the plants exhibited decreased chlorophyll content and defects in chloroplast development (Su et al., 2001; Sakuraba et al., 2013b). NYC1 and RCCR are chlorophyll catabolic enzymes and paly essential roles in chlorophyll degradation (Kusaba et al., 2007; Pruzinská et al., 2007; Sakuraba et al., 2013a). It is unexpected to find that the expression levels of two *CHL2* genes (*BnaA02G0278200NO* and *BnaA09G0127700NO*) in the white leaves were significantly higher than those in the green leaves. CHL is considered to be a rate-limiting enzyme that participates in chlorophyll degradations (Harpaz-Saad et al., 2007). However, some evidence suggested that CLH2 is not essential for chlorophyll breakdown during senescence in plans (Schenk et al., 2007; Hu et al., 2015). Overall, Chlorophyll metabolism is a complex process in *B. napus*, and the function of CHL2 in chlorophyll degradations needs to be investigated in further experiments.

Protein complexes, PSI, PSII, cytochrome b6/f complex, photosynthetic electron transport chain, and light-harvesting complexes, are embedded in thylakoid membrane and responsible for photosynthesis (Allen et al., 2011). Light energy is captured by light-harvesting complexes, and then transferred to PSI and PSII (Goral et al., 2012). Our results showed that a defect in chlorophyll accumulation led to reduced photosynthesis, which was consistent with previous research (Kim et al., 2009). Thus, we speculate that *rpoA*, *GUN1*, some specific ribosomal and PPR genes may regulate chloroplast biogenesis and development, while *CHLM*, *CHLD*, *CHLP*, *HEMF*, *DVR*, *POR*, *NYC1*, and *RCCR* participate in chlorophyll metabolism in *B. napus*. Changes in the above genes expression may directly affect photosynthesis and result in the albino phenotype in W7105.

## Data availability statement

The datasets presented in this study can be found in online repositories. The names of the repository/repositories and accession number(s) can be found at: https://www.ncbi.nlm.nih.gov/, PRJNA858621, PRJNA846709.

## Author contributions

JW and SY conceived the experiments and wrote the manuscript. JY, JL, and XM performed the experiments. YH and LZ analyzed the data. CM, JT, JS, and TF provided suggestions for designing the study. All authors contributed to the article and approved the submitted version.

## Funding

This research was funded by China Agriculture Research System of MOF and MARA (CRS12).

## Conflict of interest

The authors declare that the research was conducted in the absence of any commercial or financial relationships that could be construed as a potential conflict of interest.

## Publisher’s note

All claims expressed in this article are solely those of the authors and do not necessarily represent those of their affiliated organizations, or those of the publisher, the editors and the reviewers. Any product that may be evaluated in this article, or claim that may be made by its manufacturer, is not guaranteed or endorsed by the publisher.

## Supplementary material

The Supplementary material for this article can be found online at: https://www.frontiersin.org/articles/10.3389/fpls.2022.994616/full#supplementary-material

## Footnotes

^1^ http://cbi.hzau.edu.cn/rape/download_ext/no2127.genome.fa

^2^ http://cbi.hzau.edu.cn/rape/download_ext/no2127.genome.fa

^3^ http://www.blast2go.com/

^4^ http://www.geneontology.org/

^5^ http://www.genome.jp/kegg/genes.html
