# Supplementary material for: Bulk segregant analysis-sequencing and RNA-Seq analyses reveal candidate genes associated with albino phenotype in Brassica napus
Source: Front Plant Sci. 2022 Sep 2;13:994616. doi: 10.3389/fpls.2022.994616 (PMC9478516; doi:10.3389/fpls.2022.994616)
Supplement: Supplementary file 1 [file Data_Sheet_1.docx]

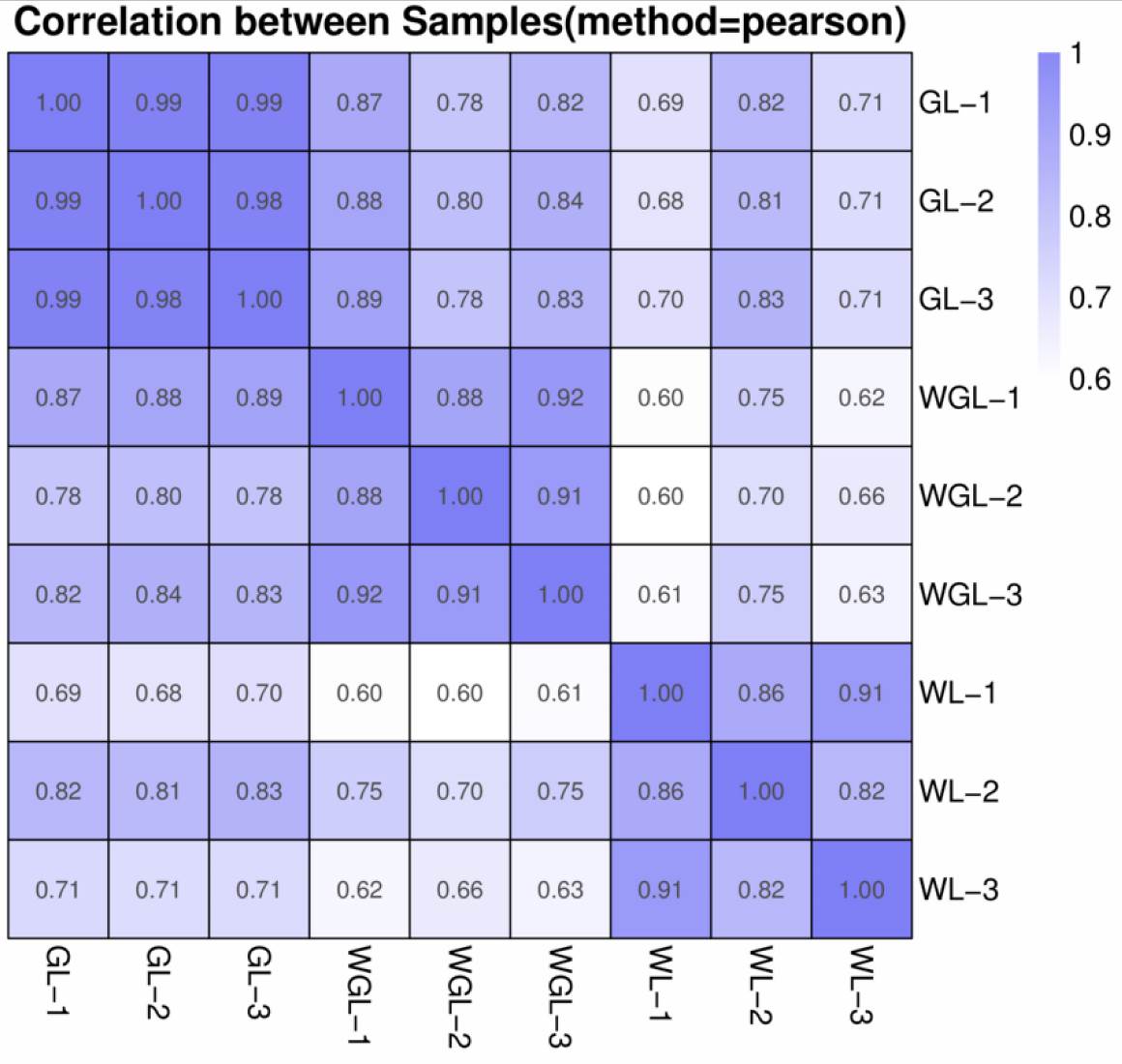


**Figure S1.** Pearson correlation coefficient among counts of transcriptome data. Numbers 1, 2 and 3 behind the GL, WGL and WL represent the biological replicates.


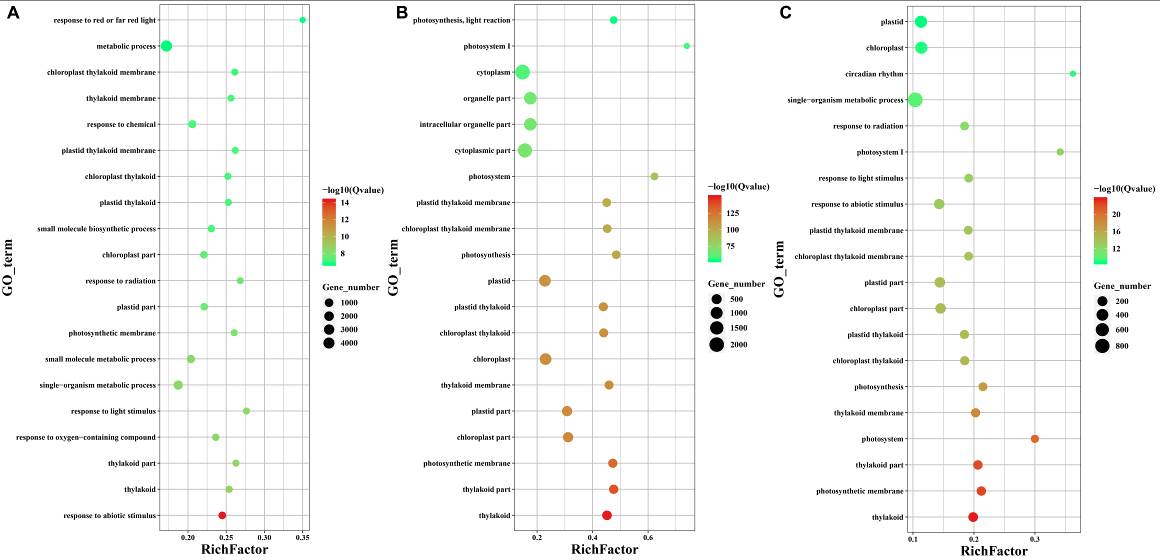


**Figure S2.** Top 20 enriched GO terms of DEGs were identified in various comparisons. **(A)** GL vs. WL; **(B)** GL vs. WGL; **(C)** WGL vs. WL.





**Figure S3.** Enriched KEGG pathways of DEGs were identified in the different comparisons. **(A)** Enriched KEGG pathways of up-regulated genes in GL vs. WGL. **(B)** Enriched KEGG pathways of down-regulated genes in GL vs. WGL. **(C)** Enriched KEGG pathways of up-regulated genes in GL vs. WL. **(D)** Enriched KEGG pathways of down-regulated genes in GL vs. WL. **(E)** Enriched KEGG pathways of up-regulated genes in WGL vs. WL. **(F)** Enriched KEGG pathways of down-regulated genes in WGL vs. WL.


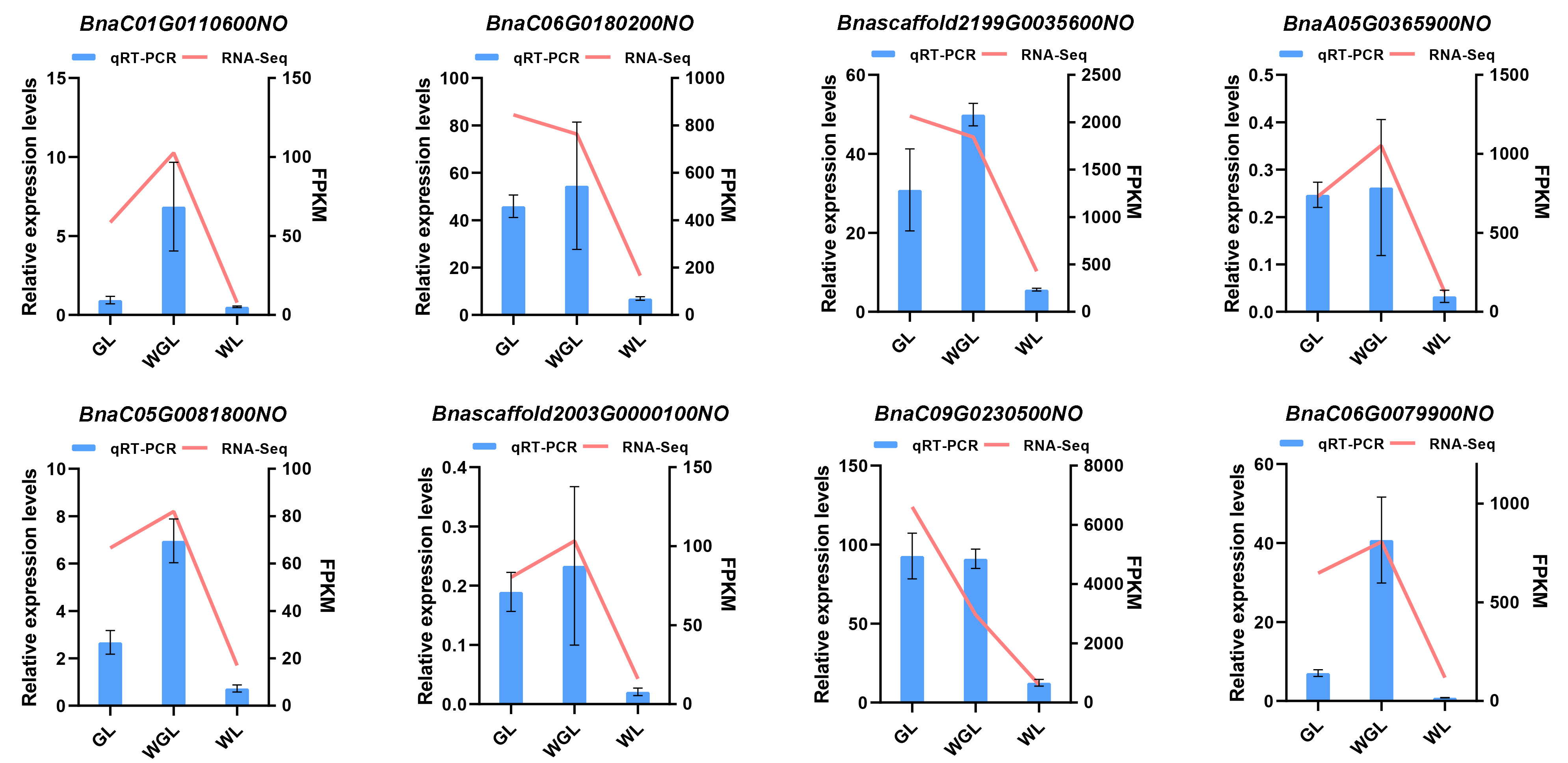


**Figure S4.** Validation of eight selected DEGs by qRT-PCR. Error bars represent the standard deviation (SD) of three biological replicates.
